# Comparison of Emergence Delirium in Patients Undergoing Laparoscopic Cholecystectomy With High or Low Fresh Gas Flow During Anesthesia: A Prospective Randomized Double‐Blind Study

**DOI:** 10.1155/anrp/5927191

**Published:** 2025-10-26

**Authors:** Sunil Bhatta, Subrata Podder, Shyam Charan Meena

**Affiliations:** ^1^ Department of Anesthesia and Intensive Care, Post Graduate Institute of Medical Education and Research (PGIMER), Chandigarh, 160012, India, pgimer.edu.in

**Keywords:** emergence delirium, fresh gas flow, lactate, RASS score, sevoflurane

## Abstract

**Context:**

Cause of “emergence delirium” (ED) after general anesthesia with volatile anesthetics in adults remains unclear. While low “fresh gas flow” (FGF) is integral to current anesthesia practice, there is limited literature about FGF’s role in ED.

**Aims:**

To compare ED in patients receiving high or low FGF during anesthesia with sevoflurane while undergoing laparoscopic cholecystectomy for gallstone disease.

**Settings and Design:**

Prospective randomized double‐blind study.

**Methods and Materials:**

One hundred American Society of Anesthesiologists (ASA) I and II patients, aged 18–50 years, were randomized to a low FGF or high FGF group of 50 patients each. After preoxygenation with 100% oxygen for 3 min, patients were induced. The low FGF group received 4 L for 5 min, followed by 1 L till the end, while the high FGF group received 4 L throughout. Sevoflurane, nitrous oxide, and oxygen (N_2_O : O_2_ = 1 : 1) were used to maintain minimum alveolar concentration (MAC) value of ≥ 1.2. A blind independent observer assessed ED by Richmond agitation‐sedation scale (RASS).

**Statistical Analysis Used:**

Student’s *T*‐test, chi‐square test, and Mann–Whitney *U* test.

**Results:**

Postoperative lactate was significantly higher in the low FGF group compared with the high FGF group (1.49 ± 0.69 vs. 1.05 ± 0.54 mmol/L, *p* ≤ 0.001). RASS scores were also higher with low FGF both immediately after PACU admission (median 1 [IQR 1–1] vs. −1 [IQR –2–0], *p* < 0.001) and at 10 min (median 0 [IQR 0–1] vs. 0 [IQR –1–0], *p* < 0.001), while scores at 20 and 30 min were comparable.

**Conclusions:**

ED and lactate levels were significantly higher in patients receiving low FGF compared to high FGF during anesthesia.

**Summary**

• FGF rate may affect ED.

## 1. Introduction

Emergence Delirium (ED) is an acute state of short‐lived​ cerebral dysfunction with an uncertain etiology, or it can be defined as a transient confusional state that is associated with emergence from general anesthesia. It is a common problem for anesthesia practitioners in the immediate postoperative period. ED manifests within the first 30 min of recovery from anesthesia, and is usually self‐limited (5–15 min), and often spontaneously resolves [1]. ED can lead to various hazards like increased intensity of pain, hemorrhage from the surgical site, removal of drains, dressings, urinary catheters, and endotracheal tubes, hypoxia, physical injuries from falling off a trolley, and risk to attending nurses and medical staff in the postanesthesia care unit (PACU) [2–4]. Restless recovery from anesthesia escalates the workload on the PACU staff and requires more adjunct medications (sedatives) to control agitation, which delays the discharge from PACU and results increase in healthcare costs. Attendants of the patient who witness this postanesthesia behavior may worry about the permanent sequelae of the reaction [1].

In recent years, interest in ED has increased due to the increasing popularity of inhalational agents’ sevoflurane and desflurane and their associations with ED [1]. The resurgence of interest in anesthesia with low fresh gas flow (FGF) over the past decades is primarily governed by the availability of low‐soluble anesthetic agents with higher costs [5]. The availability of advanced anesthesia machines with systems for monitoring inhaled and exhaled gas concentrations, including oxygen, that meet all prerequisites to practice safe anesthesia with low FGF is critical [6].

Ambulatory laparoscopic surgeries have rapidly evolved over the last decades, and the availability of improved minimally invasive surgical techniques has led to an emphasis on expansion of daycare surgeries [7]. The advantages of these procedures include less surgical trauma, decreased pain, fewer postoperative pulmonary complications, reduced rate of wound infection, and same‐day discharge from PACU [8]. The less soluble agents, desflurane and sevoflurane have changed the recovery characteristics, allowing earlier readiness for discharge in daycare laparoscopic procedures [7]. However, these anesthetic agents are considered responsible for ED, but the possible role played by low FGF used during the use of these anesthetic agents is not fully investigated.

So, in the present study, we hypothesized that patients receiving low FGF during anesthesia with the inhalation agent sevoflurane will have more ED compared to patients receiving high FGF during anesthesia with sevoflurane.

This prospective double blinded randomized study compared the incidence of ED in patients receiving high or low FGF during anesthesia with sevoflurane while undergoing laparoscopic cholecystectomy for gallstone disease.

## 2. Subjects and Methods

After approval of the protocol by the institutional ethics committee and taking consent from the patients, the prospective randomized controlled and double‐blinded study was conducted in the years 2018‐2019 in a tertiary care center. Hundred American Society of Anesthesiologists (ASA) 1 and 2 patients of 18–60 years of age of either sex with gallstone disease scheduled for laparoscopic cholecystectomy under general anesthesia on a daycare basis were included. Patients with hypersensitivity to anesthetic drugs, pregnancy, obesity (BMI > 30 kg/m^2^), cardiopulmonary disease, renal or hepatic dysfunction, respiratory insufficiency, hyperthyroidism or hypothyroidism, and antidepressant use were excluded from the study. The patients were randomized to receive low FGF (Group A) or high FGF (Group B) during anesthesia prior to surgery using computer‐generated random number tables. Codes were stored in sealed envelopes, and the anesthetist administering anesthesia was presented with envelopes containing the randomly assigned groups just prior to surgery. The anesthesia provider recorded all the demographic data (name, age, and sex) as well as hemodynamic parameters. An anesthesiologist blinded to group allocation recorded postoperative parameters in PACU.

All patients were asked to strictly follow the preoperative fasting and other instructions, and no sedative premedication was used. After preoxygenation with 6 L of 100% oxygen for 3 min, all patients were induced with intravenous (IV) propofol (1–2.5 mg·kg^−1^), IV fentanyl (1‐2 μg·kg^−1^) and IV atracurium (0.5 mg·kg^−1^). The airway was secured with the required size of endotracheal tube (ETT). After endotracheal intubation, controlled mechanical ventilation was established with tidal volume (TV) of 8–10 mL·kg^−1^ and rate of 14 breaths minute^−1^. The inspiratory and expiratory ratios were set at 1 : 2. All procedures were performed with the same type of circle breathing system and vaporizer under standard operating room conditions using the same model of anesthesia workstation (GE Datex Ohmeda Aespire View, General Electric, Boston, USA). Fresh soda lime was used for individual anesthetic procedures.

After endotracheal intubation, the patients in the low FGF group were first ventilated with a flow of 4 L (2 L + 2 L: O_2_ + N_2_O) for 5 min, and then the flow was reduced to 1 L (FiO_2_‐50% with N_2_O). The patients in the high FGF group were continuously ventilated with a 4 L/min flow after endotracheal intubation until the end of the procedure. Anesthetic depth was maintained throughout surgery in both groups, maintaining a minimum alveolar concentration (MAC) value of 1.2 (MAC 95) with sevoflurane, nitrous oxide, and oxygen (N_2_O : O_2_ = 1 : 1).

Patients were monitored continuously with 5 lead ECGs, pulse oximetry (SpO_2_), airway pressures, inspired O_2_ (FiO_2_), expired O_2_ (EtO_2_), inspired sevoflurane (FiSev) and expired sevoflurane (EtSev), inspired N_2_O (FiN_2_O) and expired N_2_O (EtN_2_O), and inspired carbon dioxide (FiCO_2_), and expired carbon dioxide (EtCO_2_), and MAC. Noninvasive blood pressure (NIBP), Heart Rate (HR), and other monitored parameters were checked and recorded every 5 min, starting from the time of induction until the end of anesthesia. Arterial blood gases (ABG) were analyzed in both groups within the first 5 min when both groups were ventilated with 4 L/min and at the end of anesthesia. In both groups, the concentration of sevoflurane was adjusted to maintain NIBP and HR within a 20% fluctuation below and above baseline values.

The patients were shifted to PACU after reversal with neostigmine and glycopyrrolate, fulfilling criteria of normothermia with stable hemodynamics, moving all extremities, and maintaining a patent and clear airway with normal breathing pattern. In PACU, patients were evaluated for ED every 10 min for the first 30 min using “Richmond agitation‐sedation scale” (RASS) [9]. Pain was assessed using “visual analogue scale” (VAS) [10] every 10 min until 30 min, and rescue analgesics in the form of fentanyl and paracetamol were used for VAS of more than 3. Postoperative nausea and vomiting (PONV) were assessed every 10 min until 30 min, and ondansetron 0.1 mg/kg was used as rescue antiemetic. Patients were discharged from the recovery room after achieving a modified Aldrete score [11] greater than 9, and the duration of their PACU stay was noted.

### 2.1. Statistical Analysis

Based on a previous study by Lepouse et al. [4], an incidence of a 20% difference between groups was assumed. Sample size was calculated through the ClinCalc sample size calculator (ClinCalc, LLC, USA). A total of 98 patients were required in both groups (49 each) at 80% power and a 0.05 alpha level. Keeping in mind the dropout of samples during the study, a sample of 50 was taken in each group.

The measured continuous data were presented as mean ± SD. To check the normality of quantitative data, Kolmogorov–Smirnov test was applied. Our data for age, weight, HR, BP, and temperature were normally distributed. So, the means of two groups were compared using the Student’s *T*‐test. Qualitative categorical variables like sex, ASA status, preoperative medications, PONV, alcohol, and drug intake were described as frequencies and proportions and were compared using chi‐square test. Inspired sevoflurane (FiSev) and expired sevoflurane (EtSev), inspired N_2_O (FiN_2_O) and expired N_2_O (EtN_2_O), and inspired carbon dioxide (FiCO_2_), and expired carbon dioxide (EtCO_2_), MAC, SpO_2_ were presented as means and compared using the Student’s *T*‐test. RASS, VAS, and modified Aldrete scores were checked by Levene’s test for equality of variances and compared using Mann–Whitney *U* test. Two‐tailed statistical tests were used for all analyses. A *p* value of < 0.05 was considered significant. The statistical analysis was carried out using MS Excel (Microsoft Excel 365, Microsoft Corporation, Redmond, USA) and “Statistical Package for Social Sciences” (IBM SPSS Statistics, IBM, New York, USA, Version 22.0 for Windows).

## 3. Results

**Figure 1:**
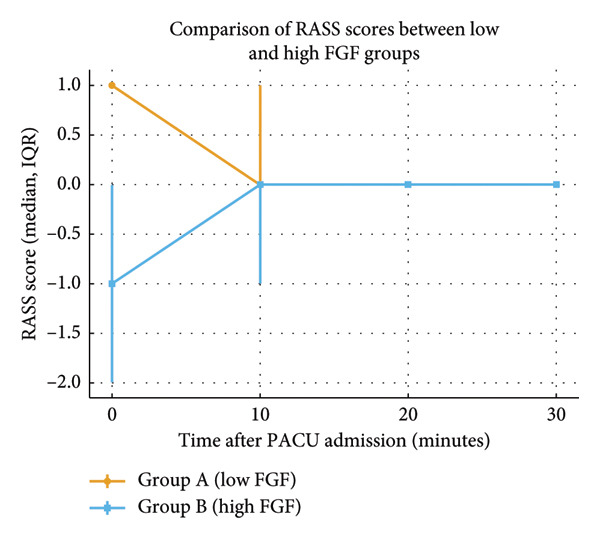
Comparison of Richmond agitation‐sedation scale (RASS) scores between low fresh gas flow (FGF) and high FGF groups at 0, 10, 20, and 30 min after admission to the postanesthesia care unit (PACU). Data are presented as median values with interquartile range (IQR) error bars. Patients in the low FGF group showed significantly higher RASS scores at 0 and 10 min compared with the high FGF group (*p* < 0.05), while scores at 20 and 30 min were comparable.

Age, sex, height, weight, BMI, ASA status, comorbidities, preoperative medications, and history of alcohol or drug abuse were comparable between the groups (Table 1). The amount of fentanyl, propofol, and atracurium used in the low FGF group and the high FGF group was comparable (Table 1). Intraoperative FiSev, EtSev, FiN_2_O, EtN_2_O, MAC, EtCO_2_, and SpO_2_ were comparable between two groups with insignificant *p* value (Table 2). Rescue analgesia with paracetamol, fentanyl, and tramadol, and PONV treatment with rescue antiemetic ondansetron and metoclopramide were comparable (Table 3). ABG analyses in low FGF and high FGF group patients taken after postinduction and before pre‐extubation were comparable, while significantly higher pre‐extubation lactate levels were noted in the low FGF group (Table 4). RASS measured at 0 and 10 min showed a significant difference between the low FGF group and the high FGF group, while RASS at 20 and 30 min intervals were comparable (Figure 1 and Table 5).

**Table 1:** Demography and inducing agents.

	Group A	Group B	*p* value
Age	40.58 ± 10.64	40.60 ± 9.01	0.99
Sex (M/F)	9/41	17/33	0.68
Height	158.79 ± 8.24	161.78 ± 6.86	0.05
Weight	63.22 ± 11.53	66.02 ± 8.52	0.17
BMI	25.34 ± 3.55	25.52 ± 3.13	0.80
ASA (I/II)	43/7	45/5	0.54
Comorbidities (yes/No)	8/42	5/45	0.37
Medications (yes/No)	7/43	5/45	0.54
Alcohol or drug abuse (yes/No)	1/49	0/50	0.32
Fentanyl	78.90 ± 18.77	83.20 ± 13.62	0.193
Propofol	97.20 ± 21.48	101.00 ± 12.66	0.284
Atracurium	30.90 ± 5.32	32.70 ± 4.97	0.083

**Table 2:** Inspired, expired sevoflurane, N_2_O, EtCO_2_, MAC, and oxygen saturation.

Group	FiSev		EtSev		FiN_2_O		EtN_2_O		MAC		EtCO_2_		SpO_2_	
	A	B	A	B	A	B	A	B	A	B	A	B	A	B
5 min	1.88 ± 0.71	2.14 ± 0.81	1.41 ± 0.45	1.60 ± 0.56	45.86 ± 5.62	45.58 ± 6.41	40.80 ± 6.84	39.90 ± 7.80	0.97 ± 0.22	0.97 ± 0.22	32.70 ± 4.06	33.10 ± 3.98	99.70 ± 0.86	99.80 ± 0.7
*p* value	0.96		0.56		0.817		0.541		1.000		0.620		0.526	
10 min	1.75 ± 0.79	1.78 ± 0.62	1.39 ± 0.54	1.41 ± 0.43	47.98 ± 5.77	47.76 ± 5.54	44.68 ± 6.04	43.78 ± 6.83	1.07 ± 0.16	1.10 ± 0.15	33.78 ± 2.99	33.30 ± 2.71	99.86 ± 0.41	99.88 ± 0.44
*p* value	0.822		0.790		0.846		0.487		0449		0.402		0.812	
15 min	1.49 ± 0.42	1.55 ± 0.45	1.27 ± 0.31	1.32 ± 0.36	49.00 ± 6.64	48.68 ± 4.72	46.16 ± 6.71	45.44 ± 5.79	1.09 ± 0.12	1.11 ± 0.16	33.78 ± 2.97	33.30 ± 2.37	99.82 ± 0.52	99.90 ± 0.36
*p* value	0.507		0.493		0.782		0.567		0.528		0.374		0.377	
20 min	1.36 ± 0.37	1.42 ± 0.39	1.20 ± 0.30	1.25 ± 0.30	49.16 ± 6.82	48.98 ± 4.29	46.72 ± 6.86	46.44 ± 4.94	1.10 ± 0.14	1.08 ± 0.13	33.86 ± 3.14	33.46 ± 2.61	99.86 ± 0.45	99.92 ± 0.34
*p* value	0.412		0.410		0.875		0.815		0.824		0.490		0.455	
25 min	1.39 ± 0.31	1.40 ± 0.44	1.21 ± 0.25	1.20 ± 0.34	48.32 ± 9.10	49.30 ± 4.00	46.96 ± 6.63	46.92 ± 4.26	1.08 ± 0.12	1.07 ± 0.15	34.18 ± 2.78	33.92 ± 2.81	99.90 ± 0.36	99.94 ± 0.31
*p* value	0.874		0.867		0.487		0.971		0.883		0.643		0.558	
30 min	1.75 ± 1.82	1.52 ± 0.51	1.32 ± 0.38	1.29 ± 0.39	49.82 ± 6.82	48.60 ± 5.59	47.18 ± 6.60	46.44 ± 5.11	1.11 ± 0.13	1.11 ± 0.15	34.68 ± 2.79	34.44 ± 2.94	99.88 ± 0.44	99.96 ± 0.28
*p* value	0.376		0.716		0.330		0.532		0.941		0.676		0.278	
35 min	1.47 ± 0.38	1.48 ± 0.35	1.29 ± 0.33	1.29 ± 0.29	50.24 ± 7.40	47.98 ± 6.58	47.62 ± 7.34	46.22 ± 3.91	1.13 ± 0.13	1.08 ± 0.17	35.10 ± 2.55	35.06 ± 2.84	99.86 ± 0.61	99.96 ± 0.28
*p* value	0.870		0.949		0.110		0.296		0.103		0.941		0.294	
40 min	1.46 ± 0.35	1.51 ± 0.39	1.28 ± 0.31	1.31 ± 0.30	50.54 ± 7.87	49.12 ± 3.94	48.28 ± 8.03	47.02 ± 3.99	1.11 ± 0.13	1.13 ± 0.12	35.56 ± 2.60	35.66 ± 2.46	99.88 ± 0.52	99.96 ± 0.28
*p* value	0.555		0.624		0.257		0.323		0.522		0.844		0.342	
45 min	1.48 ± 0.35	1.53 ± 0.39	1.29 ± 0.29	1.34 ± 0.31	50.60 ± 7.27	49.20 ± 4.05	48.38 ± 7.34	47.14 ± 4.27	1.12 ± 0.11	1.13 ± 0.09	35.68 ± 2.46	35.84 ± 2.28	99.90 ± 0.36	99.96 ± 0.28
*p* value	0.535		0.490		0.237		0.304		0.692		0.737		0.360	
50 min	1.45 ± 0.32	1.57 ± 0.43	1.29 ± 0.27	1.35 ± 0.32	50.26 ± 7.65	49.25 ± 4.01	48.10 ± 7.75	47.06 ± 4.14	1.12 ± 0.10	1.11 ± 0.09	36.26 ± 2.47	35.75 ± 2.42	99.90 ± 0.46	99.96 ± 0.20
*p* value	0.140		0.296		0.418		0.413		0.498		0.305		0.424	

**Table 3:** Postoperative rescue analgesia, PONV, and antiemetics.

		Group A	Group B	Total	*p* value
Analgesics	No analgesic	11 (22%)	15 (30%)	26	0.202
	Paracetamol	35 (70%)	35 (70%)	70	
	Paracetamol + fentanyl	1 (2%)	0	1	
	Paracetamol + tramadol	3 (6%)	0	3	

PONV	Yes	10 (20%)	4 (8%)	14	0.084
	No	40 (80%)	46 (92%)	86	

Antiemetics	No antiemetic	39 (78%)	46 (96%)	85	0.126
	Ondansetron	10 (20%)	4 (8%)	14	
	Ondansetron + metoclopramide	1 (2%)	0	1	

**Table 4:** Arterial blood gasses.

Variables	Postinduction			Pre‐extubation		
	Group	Mean ± SD	*p* value	Group	Mean ± SD	*p* value
pH	A	7.40 ± 0.05	0.970	A	7.38 ± 0.05	0.226
	B	7.40 ± 0.04		B	7.36 ± 0.04	

PaO_2_	A	204.80 ± 44.53	0.527	A	170.61 ± 51.81	0.160
	B	198.00 ± 61.17		B	157.26 ± 41.94	

PaCO_2_	A	35.96 ± 4.11	0.557	A	37.02 ± 5.26	0.222
	B	35.49 ± 3.80		B	45.32 ± 47.46	

HCO_3_	A	22.19 ± 2.10	0.217	A	21.16 ± 2.81	0.233
	B	21.00 ± 6.41		B	21.73 ± 1.90	

BE/BD	A	−1.91 ± 2.07	0.438	A	−3.59 ± 2.81	0.414
	B	−2.23 ± 2.04		B	−3.17 ± 2.12	

Lactate	A	1.20 ± 0.63	0.810	A	1.49 ± 0.69	0.001
	B	1.23 ± 0.52		B	1.05 ± 0.54	

**Table 5:** VAS score and Richmond agitation‐sedation score (RASS).

Time (minutes)	VAS			RASS		
	Group	Median (IQR)	*p* value	Group	Median (IQR)	*p* value
0	A	3 (5, 1)	0.36	A	1 (1, 1)	0.00
	B	2 (5, 0)		B	−1 (0, −2)	

10	A	3 (5, 2)	0.22	A	0 (1, 0)	0.00
	B	2.5 (5, 1)		B	0 (0, −1)	

20	A	4 (5, 3)	0.06	A	0 (0, 0)	0.08
	B	3 (4.25, 2)		B	0 (0, 0)	

30	A	3 (4, 2.75)	0.42	A	0 (0, 0)	0.32
	B	3 (4.25, 2)		B	0 (0, 0)	

VAS measured in PACU was found to be comparable between low and high FGF groups (Table 5). Modified Aldrete score from PACU and total time till discharge from PACU in low and high FGF groups were comparable (Table 6).

**Table 6:** Modified Aldrete score and duration of PACU stay.

	Group	Value	*p* value
Modified Aldrete score (median + IQR)	A	10 (10, 10)	0.171
	B	10 (10, 10)	

Duration of PACU stay (mean ± SD)	A	1.89 ± 0.61	0.747
	B	1.85 ± 0.62	

## 4. Discussion

The study compared effects of high and low FGF on ED in postoperative period in patients receiving sevoflurane as an inhalation agent. 100 ASA 1 and 2 patients were randomly allocated to a low FGF or high FGF group consisting of 50 patients each. ED was assessed using RASS immediately after arriving in the PACU and every 10 min for the initial 30 min. Other recovery characteristics measured in the PACU were pain, PONV, and postoperative vitals.

The age, weight, height, BMI, ASA status, comorbidities, and use of preoperative medications were similar in both groups. Low and high FGF groups were comparable in terms of the induction doses of fentanyl (*p* = 0.193), propofol (*p* = 0.284), and muscle relaxants (*p* = 0.83) used. Intraoperative temperature, HR, systolic blood pressure, diastolic blood pressure, mean blood pressure, oxygen saturation, fraction of inspired oxygen, end tidal oxygen concentration, fraction of inspired nitrous oxide, end tidal nitrous oxide concentration, fraction of inspired sevoflurane, end tidal sevoflurane, and MAC were comparable in both groups at different time points during the surgery.

The key finding in the present study was that sevoflurane anesthesia with low FGF intraoperatively had a higher rate of delirium in the PACU as compared to high FGF.

Muslu et al. [12] compared sevoflurane anesthesia with medium flow and low flow on postoperative cognitive function and recovery times in 36 patients undergoing laparoscopic cholecystectomy, with 18 patients in each group. They observed no significant differences in recovery times and mini‐mental state examination (MMSE) scores between anesthesia with low FGF or medium FGF groups. One hour after arrival at PACU, two patients in the medium‐flow group (11%) and three patients in the low‐flow group (17%) experienced cognitive impairment (*p* = 0.629). Three hours postoperatively, patients in both groups recovered complete normal cognitive function. They concluded that intraoperative sevoflurane consumption has no correlation to emergence recovery times or MMSE scores. According to the study, cognitive function is not altered by the FGF rate. As ED and postoperative cognitive dysfunction are not similar entities, we can only assume that sevoflurane anesthesia with low FGF and its degradation products can lead to ED. Furthermore, we assessed ED immediately upon arrival at the PACU, in contrast to the study of Muslu et al. [12], who assessed postoperative cognitive dysfunction 1 h after arrival at the PACU. In our study RASS scores showed significant variability at an early point, which implied an increased incidence of hyperactive emergence in patients receiving low FGF (*p* = 0.00,) while patients receiving high FGF were hypoactive. Hyperactive emergence excitement is associated with complications like accidental removal of catheters, IV lines, bleeding, and physical injuries. Although the RASS scores remained similar in later phases of PACU admission between the two groups, the risks associated withhyperactive excitement prevails in patients receiving low FGF.In the current study, we provided analgesia with fentanyl 1 μg/kg and induced patients with propofol and in the postoperative units, both groups of the patients have similar VAS scores, except at 20 min. Considering the findings of the Muslu et al. [12] study and our study, we can say that postoperative pain scores are not influenced by the intraoperative FGF rate. Overall, the findings from our study demonstrate similar recovery profiles and PACU stay times between both groups. However, the hyperactive emergence and its associated risks in the low FGF group are significant outcomes from the current study.

Gecaj‐Gashi et al. [13] in 2014 compared the incidence of PONV in 44 patients receiving sevoflurane anesthesia with high FGF and low FGF undergoing maxillofacial surgery. They observed the nausea and vomiting incidence in the early postoperative period of 40.9% with high FGF and 27% with low FGF. The late postoperative period demonstrated no significant differences between the groups, with 22.7% in the high‐flow group compared to 13.6% in the low‐flow group. They concluded the study by demonstrating sevoflurane anesthesia with low FGF has a low incidence of PONV and a high satisfaction rate. In the present study, 20% of the patients in the low FGF group had documented PONV in comparison to 8% of the high FGF group. Ten patients with low FGF required rescue ondansetron, and one patient required ondansetron plus metoclopramide, whereas in the high FGF group, four patients required rescue ondansetron. Although a greater number of patients with low FGF had PONV as compared to high FGF patients, the incidence is statistically insignificant. Gecaj‐Gashi et al.’s [13] finding also relates to our study, as the incidence of PONV was found to be insignificant between groups.

Fan et al. have reported that in low‐flow techniques, reducing the FGF increases the discrepancy between the O_2_ content and inspired O_2_ concentration, raising the risk of hypoxia. It is recommended that the inspiratory O_2_ concentration should be at least 30% to reliably prevent hypoxemia and ensure adequate O_2_ delivery. In our study, no decrease in inspired and expired O_2_ concentrations was observed in both groups during the operation, and no signs of hypoxia were detected in ABG analysis [14].

Tokgöz et al. [15] performed a study to compare the effects of low FGF and high FGF in children receiving desflurane anesthesia. Forty children were posted for different upper and lower limb surgeries, with 20 children in each group. They found no difference between the groups in terms of recovery hemodynamics, ABG parameters, or liver and kidney function, postoperative recovery metrics such as extubation time, orientation time, and Aldrete scores. They reported a significantly higher lactate level in the low‐flow group when compared to the high‐flow group. They concluded that, with the use of appropriate techniques and close monitoring of ABG and lactate levels, anesthesia with low FGF can be successfully performed in children. Additionally, there were no significant differences in the incidence of adverse effects like nausea and vomiting during the recovery period. The present study noticed an important increase in pre‐extubation lactate values in the low FGF group, which is similar to the study of Tokgöz et al. [15].

Previous studies have also highlighted biochemical considerations during low‐flow anesthesia. Bito and Ikeda reported that degradation products of sevoflurane may accumulate under low‐flow conditions, potentially contributing to metabolic changes [16]. Similarly, Geyik et al. observed alterations in hemodynamic parameters and lactate metabolism during low‐flow techniques [17]. While our findings of modestly higher lactate in the low FGF group are consistent with these observations, the clinical significance remains uncertain. These results should therefore be interpreted cautiously, and future studies incorporating direct markers of metabolism and inflammation are warranted.

Several factors may contribute to elevated lactate levels and ED during low‐flow sevoflurane anesthesia. Surgical stress, alterations in tissue perfusion, and metabolic effects of volatile agents are potential contributors [15, 17]. Volatile anesthetics, including sevoflurane, have been associated with glycogenolysis and subsequent lactate elevation [16]. Additionally, low‐flow anesthesia may cause subtle discrepancies between oxygen delivery and utilization, which can increase lactate even in the absence of overt hypoxia. In our study, although patients had stable hemodynamics and oxygenation, these mechanisms may partly explain the modest rise in lactate observed with low FGF. Importantly, the increase was statistically significant but biologically small, underscoring the need for cautious interpretation. Future studies incorporating inflammatory and metabolic biomarkers could clarify whether these lactate elevations reflect meaningful pathophysiological processes [18].

Patient factors such as age, cognitive function, and comorbidities can also influence lactate metabolism and ED risk, especially in older patients with preoperative cognitive impairment. However, in our study we excluded patients who were aged more than 60 years; patient with co‐existing cardiopulmonary, endocrinological, and psychiatric co‐morbid conditions were also excluded. These variables must be considered when examining the link between lactate levels and ED. The discrepancy between oxygen content and inspired oxygen concentration as observed from the Fan SZ study could explain the increased lactate level in our study. Furthermore, the increase in lactate due to accelerated glycogenolysis and neuroinflammation due to surgical stress associated with low‐flow anesthesia could account for the increase in ED in low‐FGF group in our study [14].

Avci et al. [19] performed a study in patients undergoing abdominal surgery to evaluate effects of desflurane anesthesia with low FGF compared with high FGF and its effects on perioperative hemodynamics, depth of anesthesia, and postoperative recovery. They also performed ABG analysis to see lactate and carboxyhemoglobin values. They observed that lactate values in ABG analysis in both low FGF and high FGF were not in the risky range, and lactate values between the two groups were comparable. The findings of the study differ from ours in that we observed a significant increase in pre‐extubation lactate values in the low FGF group when compared to the group receiving high FGF.

In our study, patients with low FGF developed hyperactive delirium, and patients with high FGF developed hypoactive delirium. This observation in PACU did not influence the PACU discharge time between the groups. The modified Aldrete score for PACU discharge was comparable between the two groups. Time of PACU discharge and similar modified Aldrete scores show anesthesia with low and high FGF is equally effective in daycare settings.

Based upon the findings of our study, we can say that sevoflurane anesthesia with low FGF for laparoscopic cholecystectomy showed a significantly increased incidence of ED and significantly higher levels of postanesthesia serum lactate. Our findings not only confirm the feasibility of both high‐ and low‐flow sevoflurane anesthesia but also add nuance by showing that low FGF is associated with higher ED scores and modestly elevated lactate, suggesting a potential link between anesthetic technique, metabolic response, and recovery quality.

Another limitation is that ED was assessed only during the immediate PACU period (0–30 min). This was based on existing evidence that ED is most often an early postoperative event, typically arising within the first 30 min after emergence and resolving spontaneously within 5–15 min. Since the greatest risks such as accidental removal of tubes, bleeding, or injury are concentrated in this critical window, we focused our assessment on the phase of greatest clinical relevance. Nevertheless, a longer follow‐up period might have provided additional information regarding delayed or persistent delirium, and future studies should include extended observation and formal postoperative delirium assessments [3, 4, 9]. An additional limitation is that potential confounders such as preoperative anxiety, individual pain perception, and intraoperative depth of anesthesia (BIS values) were not assessed. However, we attempted to minimize variability by using standardized anesthetic protocols, uniform analgesia, and titration of sevoflurane to a target MAC with stable hemodynamics. Although BIS can provide additional information, its routine use in laparoscopic cholecystectomy under sevoflurane anesthesia remains debated, and clinical endpoints (MAC and hemodynamics) are considered sufficient in this setting. Future studies incorporating structured anxiety measures, pain sensitivity tools, and BIS monitoring may provide further insights [3, 4, 6].

This study focused on clinically relevant outcomes and serum lactate, a practical perioperative marker. Furthermore, we did not measure neuroinflammatory biomarkers such as IL‐6 or TNF‐α, which could have clarified the mechanistic link between lactate elevation and ED. Our interpretation of lactate as a marker of neuroinflammation therefore remains speculative, and future studies should incorporate such biomarkers [20, 21].

Although the lactate increase (∼0.4 mmol/L) was statistically significant, its biological impact is likely modest. Such small changes may reflect subtle metabolic or neuroinflammatory effects rather than clinically relevant hypoperfusion. We therefore interpret lactate as a potential surrogate marker and have noted this limitation, suggesting future studies with biomarker correlation to clarify its significance [15, 18].

We acknowledge that nitrous oxide can influence postoperative outcomes such as nausea, vomiting, and cognitive recovery. In our study, however, N_2_O was used in both high and low FGF groups in the same concentration (N_2_O : O_2_ = 1 : 1), ensuring that any effect of nitrous oxide was balanced across groups. Therefore, differences observed in ED and lactate levels are unlikely to be attributable to N_2_O itself but rather to the variation in FGF. Nonetheless, we agree that the independent contribution of N_2_O to postoperative outcomes remains an important consideration, and future studies comparing low and high flow anesthesia without nitrous oxide could further clarify this issue [22, 23].

While much of the classical ED literature emphasizes pediatric populations, several adult‐focused studies and reviews report that ED is a relevant perioperative problem in adults and identify inhalational anesthesia as a consistent risk factor. Large observational work has shown an increased incidence of emergence agitation with inhalational versus IV techniques and identified multiple patient‐ and procedure‐related risk factors (age, male sex, pain, type of surgery) rather than a single causal mechanism. Recent adult reviews and prospective studies also highlight wide variability in ED incidence because of differing case definitions and assessment timing but confirm that volatile agents (including sevoflurane) are frequently implicated. Our findings of higher ED scores with low FGF sevoflurane anesthesia therefore extend adult data by linking a modifiable delivery parameter (FGF) to both early behavioral disturbance and a modest metabolic signal (lactate) and underscore the need for further adult trials that standardize ED definitions and include mechanistic biomarkers [24–26].

Our study has certain shortcomings, including being conducted in a single center with a small sample size, using sevoflurane, which is known to cause ED, and using laparoscopic cholecystectomy which is a short duration surgery. The effect of low FGF needs further studies with larger cohorts, longer follow‐up, varied anesthetic techniques, and incorporation of biomarker analysis are needed to validate and extend these findings.

## Conflicts of Interest

The authors declare no conflicts of interest.

## Author Contributions

Sunil Bhatta, Subrata Podder and Shyam Charan Meena: concepts, design, definition of intellectual content, literature search, clinical studies, experimental studies, data acquisition, data analysis, statistical analysis, manuscript preparation, manuscript editing, manuscript review, and guarantor.

## Funding

No sources of funding/support received for the study.

## Data Availability

Data described to support the findings is easily accessible in the article.
